# Weight‐Lowering Drugs and Natural Female Fertility—A Systematic Review and Meta‐Analysis

**DOI:** 10.1111/cob.70092

**Published:** 2026-06-17

**Authors:** Shaikha Jabor Alnaimi, Dana Muwafag Alsugeir, Li Wei, Kirsten Harvey, Ruth Brauer

**Affiliations:** ^1^ Research Department of Practice and Policy, School of Pharmacy University College London London UK; ^2^ Pharmacy Department, Hamad bin Khalifa Medical City Hamad Medical Corporation Doha Qatar; ^3^ Department of Pharmacy Practice, College of Pharmacy Imam Abdulrahman bin Faisal University Dammam Saudi Arabia; ^4^ Research Department of Pharmacology, School of Pharmacy University College London London UK

**Keywords:** female fertility, pregnancy, weight‐lowering drugs, women's health

## Abstract

Overweight and obesity are global health concerns linked to impaired female fertility. Weight‐lowering drugs are an alternative for achieving weight loss; however, their effect on natural female fertility is unclear. A systematic review and meta‐analysis were conducted to summarise the literature on the effects of weight‐lowering drugs on ovulation, conception, pregnancy and live birth rates. Inclusion criteria comprised interventional and observational studies involving women with overweight or obesity receiving weight‐lowering drugs, compared with non‐users, lifestyle modifications or other medications. MEDLINE, Embase, CINAHL, CENTRAL and ClinicalTrials.gov were searched, yielding 2731 records. After screening, seven clinical trials were included (*n* = 575), six of which were randomised. Sample sizes ranged from 40 to 120 women aged 25.9–29.7 years. Six trials evaluated orlistat, while one assessed semaglutide. In four trials, orlistat was associated with a higher ovulation rate than lifestyle modifications. A meta‐analysis of ovulation rates comparing orlistat and metformin showed no significant difference (RR = 0.78, 95% CI: 0.41–1.49; *p* = 0.45). Two studies reported pregnancy rates: one found a higher rate with orlistat compared to lifestyle modifications (23.3% vs. 6.7%, *p* = 0.044), and another showed that adding semaglutide to metformin increased the pregnancy rate compared to metformin alone (35% vs. 15%, *p* < 0.05). Future studies should address current limitations and evaluate the effects of newer weight‐lowering drugs on natural pregnancy and live birth rates in adequately powered studies.

## Key Points

- What is already known about this subject?
  ○ Obesity is associated with impaired female fertility, mainly due to insulin resistance, hormonal imbalance and inflammation.
  ○ Weight‐lowering drugs are used to help women with obesity achieve weight loss, but their effects on natural fertility outcomes are uncertain.
  ○ Previous systematic reviews have mainly focused on lifestyle interventions.
- What this study adds?
  ○ This is the first systematic review and meta‐analysis summarising evidence on weight‐lowering drugs and natural fertility outcomes in women with overweight or obesity.
  ○ Orlistat appears to improve ovulation rates compared with lifestyle modification but not when compared with metformin; however, higher‐quality studies are needed to confirm these findings.
  ○ Limited evidence suggests that semaglutide may enhance natural pregnancy rates, warranting larger studies.

## 1 Introduction

A report by the Non‐Communicable Disease Risk Factor Collaboration published by *the Lancet* estimated the prevalence of underweight and obesity in a pooled sample with more than 200 million adults and children from 200 countries and territories [1]. These estimates are based on high‐quality studies meeting predefined inclusion criteria and are evaluated for population representativeness. The authors report that the global age‐standardised obesity rates in women have increased from 8.8% in 1990 to 18.5% in 2022, and remained higher than that in men at both timepoints [1]. A well‐established consequence of excessive body weight in women is impaired fertility [2]. Given the increasing trends in female obesity, a potential rise in the number of women seeking medical care for impaired fertility can be anticipated. The National Institute for Health and Care Excellence (NICE) guidelines recommend the use of weight‐lowering drugs for people with obesity who are not able to achieve target weight loss with lifestyle modifications and have weight‐related comorbidities [3]. Prior to the advent of glucagon‐like peptide‐1 receptor agonists (GLP‐1 RAs), beginning with liraglutide in 2017 in the United Kingdom (UK), obesity pharmacotherapy was limited to a few medications, and many new drugs struggled with safety and efficacy issues [4, 5]. Since its approval in the United Kingdom in 1998, the lipase inhibitor orlistat remained the preferred therapeutic alternative at the time given its efficacy and acceptable tolerability [6, 7].

Other weight‐lowering drugs include phentermine, lorcaserin, naltrexone/bupropion, sibutramine and phentermine/topiramate. The centrally acting phentermine acts as an appetite suppressant; however, it is not recommended for long‐term use due to abuse potential [8]. Lorcaserin produces weight‐lowering effects by suppressing appetite through serotonin 2C receptor agonist activity. In 2022, however, it was withdrawn from the United Kingdom markets due to its association with an increased risk of malignancies [8]. The combination of naltrexone/bupropion decreases weight by suppressing appetite at the hypothalamic level and is associated with adverse psychiatric outcomes including depression and increased suicidality [8]. Sibutramine is a serotonin–norepinephrine reuptake inhibitor (SNRI) and was used as an appetite suppressant before it was withdrawn by the European Medicine's Agency in 2010 due to cardiovascular safety concerns including increased risk for myocardial infarction and stroke [9]. Finally, phentermine/topiramate is another combination where phentermine acts as an appetite suppressant while topiramate contributes additional weight loss benefits through energy regulation. However, this combination is associated with neurological adverse effects such as impaired attention and paraesthesia [10].

Given these unmet safety needs with most weight‐lowering drugs, the introduction of GLP‐1 RA has resulted in a paradigm shift in obesity pharmacotherapy. Their approval and subsequent inclusion in clinical guidelines were driven by landmark trials demonstrating both efficacy and safety [3, 11]. Currently, the GLP‐1 RAs recommended by the NICE guideline for weight loss are liraglutide and semaglutide, in addition to tirzepatide, a dual acting glucose‐dependent insulinotropic polypeptide (GIP)/GLP‐1 RA [3].

Previous systematic reviews evaluated the impact of various weight loss strategies on female fertility [12, 13]. In a systematic review by Sim et al., it was found that weight reduction achieved by dietary and lifestyle modifications, the use of intragastric balloons or bariatric surgery resulted in an increase in pregnancy rates among women undergoing assisted reproductive technology (ART) [13]. Another meta‐analysis that examined the influence of weight‐loss interventions on fertility in both women and men incorporated a range of diet and lifestyle interventions; however, it had greater emphasis on lifestyle modifications [14]. Currently, there is no systematic review focusing on investigating the effect of weight‐lowering drugs on spontaneous fertility outcomes in women with overweight and obesity. Therefore, the aim of this review is to synthesise the evidence on natural female fertility outcomes including ovulation, conception, pregnancy and live birth rates associated with the use of weight‐lowering drugs.

## 2 Materials and Methods

A predefined clinical question was developed following the population, intervention, comparator, outcomes and study design (PICOS) format. We defined the population as women with overweight or obesity. The interventions included weight‐lowering drugs, while the comparators were non‐users, lifestyle modifications, other weight‐lowering drugs or metformin. The outcomes of interest were ovulation rate, conception rate, pregnancy rate and live birth rate. Study designs were interventional trials or observational studies. Case studies, systematic literature reviews, descriptive studies (without a comparator), animal studies and pharmacokinetic and pharmacodynamic studies were excluded. In addition, women undergoing ART were excluded. The protocol of this review was pre‐registered with the International Prospective Register of Systematic Reviews (PROSPERO): CRD42024597638 (October 2024) and follows the Preferred Reporting Items for Systematic Reviews and Meta‐Analyses (PRISMA) recommendations [15].

The databases searched were Medline, EMBASE, Cochrane Central Register of Controlled Trials (CENTRAL) and Cumulative Index to Nursing and Allied Health Literature (CINAHL). ClinicalTrials.gov was also searched for results of unpublished trials. The search was limited to English language and human studies. All databases were searched on the 1st of October 2025 from inception. The detailed search strategy is presented in (Tables S1–S5).

**FIGURE 1:**
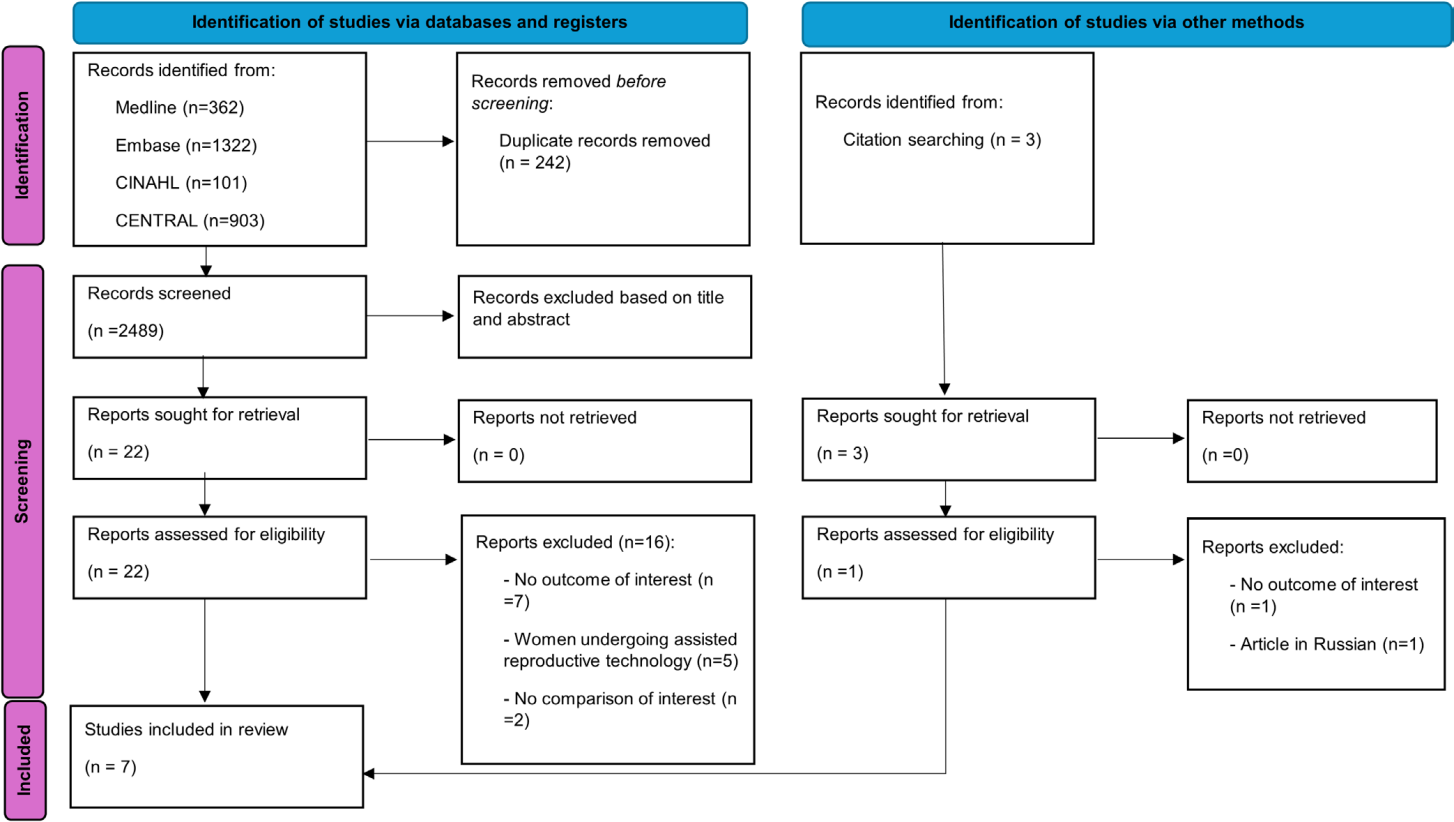
PRISMA diagram.

A PRISMA flow chart (Figure 1) and an online tool (Rayyan.ai https://rayyan.ai/) were used to screen the literature. Initially, all generated reports were screened based on titles and abstracts by S.J.A. and D.M.A. The full text of the resulting articles was then screened in a second round by S.J.A. and D.M.A. to identify eligible studies. Disagreements were resolved by discussion and consensus among the reviewers or by discussion and consensus with other reviewers (K.H. and R.B.). A data extraction tool was developed using Microsoft Excel to collect study information. Collected data included first author's name, publication year, country, study design, follow up, sample size, study period, population, number of participants per group, type of intervention/exposure, type of control, treatment duration, outcomes, mean age, mean body mass index (BMI) and outcomes reported.

### 2.1 Quality Assessment

Version 2 of the Cochrane risk‐of‐bias tool for randomised trials (RoB 2) was used to assess the quality and risk of bias in the included studies [16]. After completing the five domains, RoB 2 generates an overall assessment of the risk of bias, classified as ‘low risk’, ‘some concerns’ and ‘high risk’. For observational studies, Risk Of Bias In Non‐randomised Studies—of Interventions (ROBINS‐1) was planned to be used for observational studies but ultimately not needed. S.A. and D.A. independently assessed the quality of the studies.

### 2.2 Synthesis of Results

A meta‐analysis was originally planned, including the generation of forest plots using a random‐effects model. Where sufficient data were available, subgroup analyses by polycystic ovary syndrome (PCOS) status were planned. For each outcome, a minimum of three studies were required to perform a quantitative synthesis and generate a forest plot. Heterogeneity among studies was to be assessed using the *I* ^2^ statistic, with a value *I* ^2^ > 50% indicating significant heterogeneity. Assessment of publication bias was planned using Egger's test, in addition to a visual inspection of funnel plots. For studies reporting outcomes as proportions only, the percentages, relative risks (RRs) and 95% confidence interval (95% CI) were calculated and reported. To facilitate the determination of study eligibility for synthesis, study characteristics (e.g., interventions, comparators) and outcomes were tabulated using Microsoft Excel. Where meta‐analysis was not feasible due to insufficient or heterogeneous data, a narrative synthesis was performed. Studies were grouped according to intervention and outcome, and findings were summarised by comparing the direction and magnitude of effects across studies.

## 3 Results

### 3.1 Study Selection

The systematic search identified 2255 articles (Figure 1). After removing duplicates, 2018 articles were screened by title and abstract, resulting in the exclusion of 1998 records. Twenty articles were retrieved for full‐text review, with 100% agreement between reviewers. Following the full‐text screening, five articles were deemed fully eligible and were included in this review. In addition, a manual citation search was done, and three further articles were identified, of which one was included in the final selection of papers. In total, seven articles were included in the review [17, 18, 19, 20, 21, 22, 23]. Reasons for the exclusion of full‐text articles were mainly related to outcomes (i.e., did not include any of our pre‐specified outcomes) [24, 25, 26, 27, 28, 29, 30], or the population was defined as women undergoing ART [31, 32, 33, 34, 35].

### 3.2 Study Characteristics

The details of the seven eligible studies are presented in (Table 1). All of the studies were clinical trials and were published from 2009 to 2025. Among these studies, five were from Asia, one from Egypt and one from the United Kingdom. The sample sizes in these studies ranged from 40 to 120 women, with average ages between 25.9 and 29.7 years. Six studies assessed orlistat as a weight‐lowering drug, while one study [23] evaluated semaglutide combined with metformin. Most studies had a follow up of 3 months, except the work of Al‐Qahwajy et al. who followed participants for 6 months [17] and Chen et al. who followed participants for approximately 9 months [23].

**TABLE 1:** Characteristics of the selected studies.

Author	Year	Country	Study design	Follow up (months)	Sample size	Age (years), mean (SD)	BMI (kg/m^2^), mean (SD)	Intervention	Comparator/s
Al‐Qahwajy et al.	2022	Egypt	Clinical trial	6	120	29.18 (4.42)	30.64 (3.38)	Orlistat	Lifestyle modifications
Chen et al.	2025	China	Clinical trial	9.3	80	28.5 (4.5)	28.0 (2.7)^a^	Semaglutide + metformin	Metformin
Ghandi et al.	2011	Iran	Clinical trial	3	80	27 (4.92)	33.69 (4.23)	Orlistat	Metformin
Kumar et al.	2014	India	Clinical trial	3	90	Not reported	Not reported	Orlistat	Lifestyle modifications Metformin
Metwally et al.	2009	United Kingdom	Clinical trial	3	40	29.70 (5.80)	39 (4.45)	Orlistat	Metformin
Munir et al.	2018	Pakistan	Clinical trial	3	45	25.89 (5.35)	33.03 (5.3)	Orlistat	Lifestyle modifications
Rahman et al.	2017	Bangladesh	Clinical trial	3	120	26.75 (4.66)	30.63 (2.18)	Orlistat	Lifestyle modifications

Abbreviations: BMI, body mass index; SD, standard deviation.

^a^ Chen et al. reported BMI as median (IQR); converted to approximate mean (SD) for consistency.

### 3.3 Narrative Synthesis

All six studies reported ovulation rates, while conception rates were reported by three studies, and only one study reported pregnancy rates [17]. None of the studies reported live birth rates. Three of the studies evaluated orlistat against lifestyle modifications [17, 21, 22], two compared orlistat with metformin [18, 20] and one study included three groups comparing orlistat, metformin and lifestyle modifications [19]. One study compared semaglutide combined with metformin versus metformin alone [23]. In studies that compared orlistat to lifestyle modifications, ovulation was the only outcome reported in more than three studies. Similarly, in studies comparing orlistat with metformin, ovulation was the only outcome reported by three studies. Across all comparisons, the ovulation rate was higher in the orlistat group than in the comparator groups, except in two studies where ovulation was higher in the metformin group [18, 20].

Conception rate was reported by Kumar et al., Metwally et al. and Munir et al. [19, 20, 21]. Kumar et al., the conception rates for the orlistat, metformin and lifestyle modification groups were 40%, 26.7% and 3.3%, respectively (*p* = 0.003) [19]. On the other hand, Metwally et al. report conception rates of 5% in the orlistat group and 25% in the metformin group, with no statistically significant difference (*p* > 0.10) [20]. Munir et al. reported conception rates of 6.7% in the orlistat group and 0% in the lifestyle modification group. However, in this study, conception rate was not predefined and the method of outcome assessment was not reported [21].

Pregnancy rates were reported by two studies. Al‐Qahwajy et al. reported a pregnancy rate of 23.3% in the orlistat group compared to 6.7% in the lifestyle modifications group (*p* = 0.044) [17]. Chen et al. reported natural pregnancy rates of 35% in the semaglutide plus metformin group compared to 15% in the metformin alone group (*p* < 0.05) [23]. The details of the outcomes of each study are presented in (Table 2).

**TABLE 2:** Outcomes of the selected studies.

Author	Year	Population	Intervention	Comparator(s)	Post‐treatment BMI (kg/m^2^), mean (SD)	Ovulation (%), RR (95% CI)	Conception (%), RR (95% CI)	Pregnancy (%), RR (95% CI)
Al‐Qahwajy et al.	2022	Overweight and obesity and primary infertility	Orlistat	Lifestyle modifications	Not reported	100% vs. 80% 1.25 (1.10–1.42)	Not reported	23.3% vs. 6.7% 3.50 (1.22–10.02)
Chen et al.	2025	Overweight and obesity with PCOS	Semaglutide + metformin	Metformin	Semaglutide + MET: 25.63 (24.28, 27.74)^b^ Metformin: 27.25 (25.59, 28.52)^b^	Not reported	Not reported	35% vs. 15% 2.33 (1.05–5.19)
Ghandi et al.	2011	Obesity and PCOS	Orlistat	Metformin	Orlistat: 33.24 (4.19) Metformin: 31.03 (3.43)	15% vs. 30% 0.50 (0.2–1.20)	Not reported	Not reported
Kumar et al.	2014	Overweight and obesity with PCOS	Orlistat	Lifestyle modifications Metformin	Not reported	Orlistat: 33.3% Lifestyle modifications: 3.3% Metformin: 23.3% Orlistat vs. lifestyle modifications: 10 (1.36–73.33) Orlistat vs. metformin: 1.43 (0.63–3.25)	Orlistat: 40% Lifestyle modification: 3.3% Metformin: 26.7% Orlistat vs. lifestyle modifications: 12 (1.66–86.59) Orlistat vs. metformin: 1.5 (0.72–3.14)	Not reported
Metwally et al.	2009	Obesity and anovulation	Orlistat	Metformin	Orlistat: 37.6 (4.9) Metformin: 37.7 (5.36)	25% vs. 40% 0.63 (0.25–1.58)	5% vs. 25% 0.20 (0.03–1.56)	Not reported
Munir et al.	2018	Obesity and PCOS	Orlistat	Lifestyle modifications	Orlistat: 30.65 (5.0) Lifestyle modifications: 30.95 (5.08)	60% vs. 6.7% 9.00 (1.32–61.14)	6.7% vs. 0%^a^	Not reported
Rahman et al.	2017	Overweight or obesity and subfertility	Orlistat	Lifestyle modifications	Orlistat: 29.04 (2.75) Lifestyle modifications: 28.61 (1.57)	61.7% vs. 45% 1.37 (0.97–1.93)	Not reported	Not reported

Abbreviations: BMI, body mass index; CI, confidence interval; PCOS, polycystic ovarian syndrome; RR, relative risk; SD, standard deviation.

^a^ Not estimable due to zero events in comparator group.

^b^ Post‐treatment BMI for Chen et al. reported as median (IQR).

Among the included studies, four studies restricted their population to women with PCOS [18, 19, 21, 23]. Among these, ovulation rates were higher with orlistat compared with lifestyle modifications in Kumar et al. and Munir et al. [19, 21]. In comparisons of orlistat with metformin, Kumar et al. reported higher ovulation rates with orlistat, whereas Ghandi et al. reported higher rates with metformin [18, 19]. Additionally, Kumar et al. and Munir et al. both reported higher conception rates with orlistat compared with lifestyle modifications [19, 21]. Chen et al. was the only study reporting pregnancy outcomes in a PCOS population, demonstrating higher rates with semaglutide plus metformin compared with metformin alone [23].

**FIGURE 2:**
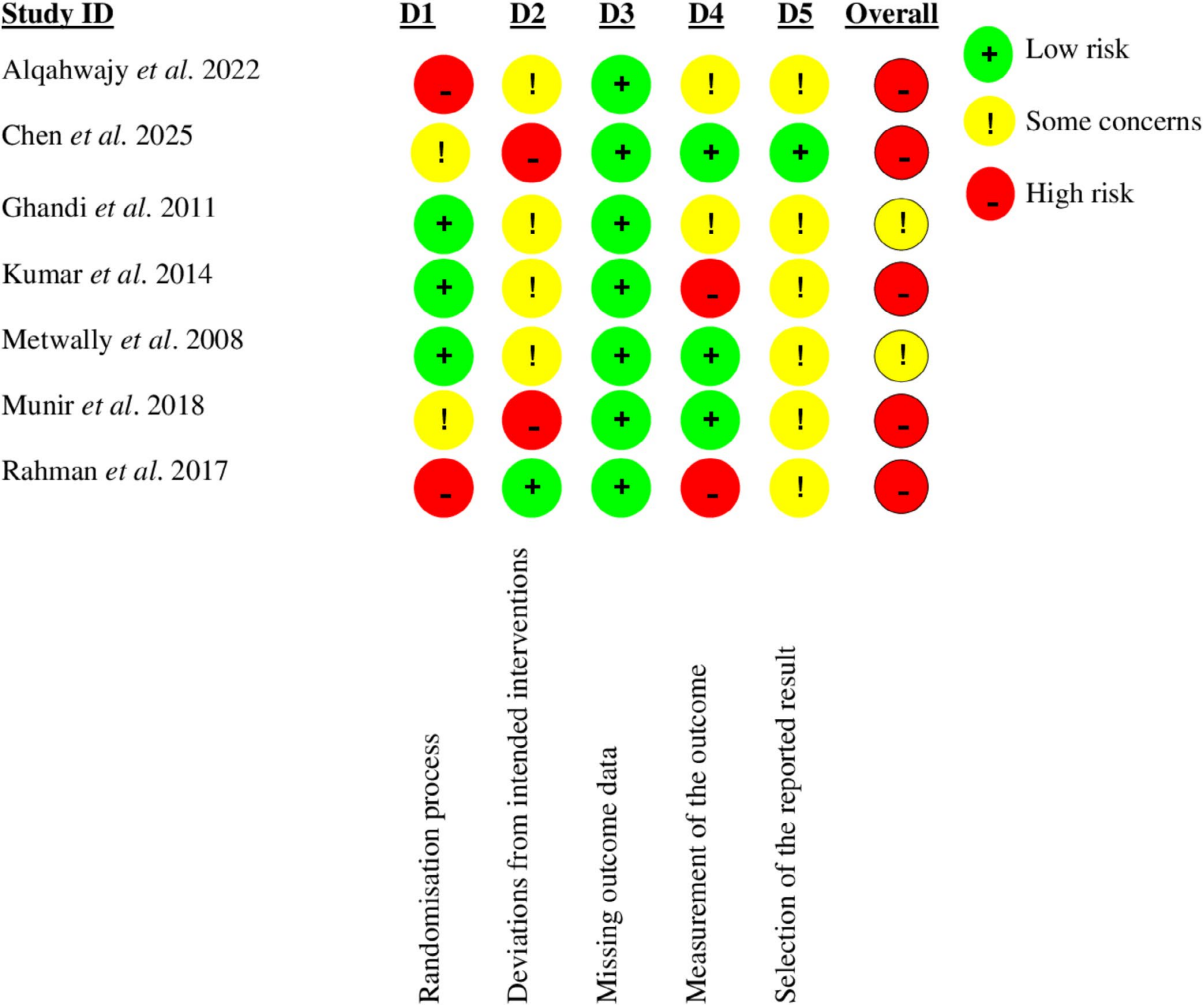
Quality assessment using version 2 of the Cochrane risk‐of‐bias tool for randomised trials (RoB 2).

The risk of bias using the RoB 2 tool is presented in (Figure 2). Five studies included in the review had a high risk of bias (*n* = 5), and the remaining studies had some concerns of bias (*n* = 2). The main factors contributing to the high risk of bias were the method of randomisation, allocation concealment and outcome assessment. The primary sources of potential bias were the open‐label study design and insufficient information regarding blinded outcome assessment.

### 3.4 Meta‐Analysis (Quantitative Analysis)

Based on the number of studies identified with a focus on ovulation rates, two meta‐analyses were planned. One meta‐analysis compared orlistat versus lifestyle modifications, and another meta‐analysis compared orlistat and metformin. A high risk of bias was identified in all four studies focussed on orlistat versus lifestyle interventions. Therefore, the results of this meta‐analysis, comprising 345 participants, were considered unreliable (Figure S1).

**FIGURE 3:**
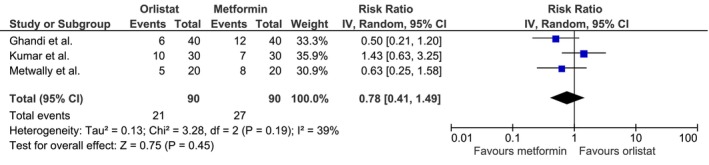
Forest plot of the effect of orlistat versus metformin on ovulation.

The second meta‐analysis comprised three studies that compared the ovulation rate between orlistat and metformin users with a total sample of 180 participants. The results show that there was no statistically significant difference in ovulation rate between orlistat users and metformin users (RR = 0.78, 95% CI: 0.41–1.49; *p* = 0.45). The test of heterogeneity showed an *I* ^2^ = 39% indicating an acceptable level of heterogeneity across studies. The results of the comparison are presented in (Figure 3). Subgroup analysis for women with PCOS was not feasible, as both Ghandi et al. and Kumar et al. restricted their inclusion to this population, and 87.5% of participants in Metwally et al. had a diagnosis of PCOS, limiting the ability to perform stratified analysis.

## 4 Discussion

This systematic review presents a summary of the literature on the role of weight‐lowering drugs on female fertility. Ovulation rates were consistently reported across all studies, while conception and pregnancy rates were less frequently reported. No studies reported live birth rate. The results point toward a potential benefit of orlistat over lifestyle modifications, although the quality of evidence was limited due to the high risk of bias in the majority of included studies. When orlistat was compared with metformin, there was no statistically significant difference between the two interventions. Conception rates appeared higher with orlistat compared to lifestyle modifications, whereas findings were inconsistent when orlistat was compared to metformin. Six studies included in this review assessed the effect of orlistat on female fertility, while one recent study evaluated a GLP‐1 RA (semaglutide) combined with metformin. Moreover, all studies were small clinical trials and the wide CIs around the effect estimates were indicative of most trials being underpowered. In addition to ovulation rate and conception rate, two studies assessed the clinically relevant outcome of pregnancy rate [17, 23].

This review presents the first systematic review of the effect of weight‐lowering drugs on natural fertility in women with obesity that is not limited to those with PCOS. Previous systematic reviews and meta‐analyses evaluated female fertility using different interventions and outcome measures. Boyle et al. conducted a meta‐analysis on the effect of dietary interventions on fertility in women with obesity [36]. The sample included 5938 women from 27 randomised controlled trials (RCT) who were followed for at least 1 year after diet‐based weight loss interventions. However, the study did not find a statistically significant difference in pregnancy rates between the intervention and control groups. The authors describe an under‐reporting of fertility outcomes as a limitation in studies assessing weight loss interventions in women with obesity. This is similar to this review where the majority of studies reported ovulation rate rather than endpoints that are more relevant to women seeking conception (i.e., pregnancy and live birth rates). This was also reported in another recent meta‐analysis that assessed the effect of insulin sensitisation on metabolic and fertility outcomes in women with overweight and obesity [37]. Unlike this study, their inclusion criteria were restricted to women with PCOS and a double‐blind study design. The pooled analysis assessed several insulin sensitisers on BMI change, fasting blood glucose, menstrual frequency, hormonal profile and pregnancy. Despite the significant findings in favour of insulin sensitisers use in improving the metabolic and hormonal profiles, the lack of pregnancy data emphasises the apparent gap in the literature on the effects of obesity pharmacotherapy on reproductive outcomes.

In terms of other drugs producing weight‐lowering effects, several studies have compared the effects of orlistat and metformin in women. A head‐to‐head RCT comparing their effect on body composition in premenopausal women reported that orlistat produced a greater weight loss and a lower fat mass compared to metformin, while insulin resistance was improved in both arms with no statistically significant difference [38]. A meta‐analysis of studies comparing the two drugs in terms of weight loss and metabolic and hormonal profiles in women with PCOS, both drugs produced similar favourable outcomes in terms of weight loss, insulin resistance and testosterone levels [39]. The results of these studies may explain the findings of our meta‐analysis, in which no difference was found in ovulation rate between women prescribed metformin versus orlistat. Metformin is an anti‐diabetic drug that produces glucose‐lowering effects by improving insulin sensitisation and decreasing hepatic glucose production [40]. It is not approved for weight loss but may be prescribed off‐label to improve ovulation in women with PCOS [41]. This indication is based on studies comparing metformin to placebo, where metformin was associated with an improvement in ovulatory function in women with PCOS [42, 43]. However, in studies comparing metformin versus clomiphene, clomiphene was superior to metformin in improving pregnancy rates and live birth rates [44, 45]. In light of this evidence, the 2023 international evidence‐based guideline for the assessment and management of PCOS now recommends clomiphene in preference to metformin in women with anovulatory infertility [41].

In this review, studies assessing weight‐loss interventions in women undergoing ART were excluded. This is because these women undergo standardised ovarian stimulation, hormonal therapy and embryo transfer procedures which bypass the natural fertility processes affected by increased weight. Moreover, pregnancy outcomes can be affected by external factors such as the laboratory conditions making it difficult to ascertain the effect of weight‐lowering drugs in this population [46]. It is well established that women with obesity undergoing ART are at an increased risk for unfavourable pregnancy outcomes such as lower conception rates, higher gonadotropin requirements and an elevated risk of pregnancy loss. Furthermore, women who successfully become pregnant are at increased risk for pre‐eclampsia, gestational diabetes and caesarean delivery [47]. Clark et al. evaluated the effect of weight loss with lifestyle interventions in women with obesity undergoing fertility treatment [48]. Prior to the programme, women with obesity had a miscarriage rate of 75%, and following a 6‐month programme consisting of dietary modifications and exercise, the post‐intervention miscarriage rate was 18% in the same women. This was reflected in the healthcare costs of fertility treatment, as before the programme the cost of fertility treatment was $550 000 Australian dollars for two live births in 67 women, while after the treatment the cost was $210 000 for 45 live births in the same number of women. In addition to lifestyle modifications, some studies assessed the effect of weight‐lowering drugs in women undergoing fertility treatments. In a recent retrospective study by Tong et al., women undergoing ART who received orlistat were compared to matched comparators who received standard care without weight‐lowering drugs. By the end of follow up, the orlistat group had higher pregnancy rates (59.46% versus 39.47%, *p* = 0.004) [33]. However, there was no difference in live birth rates. Similarly, a large double blind RCT of 877 women did not find a difference in live birth rates between orlistat and placebo [34]. In addition to orlistat, liraglutide has been evaluated as a pre‐treatment intervention before ART. A pilot RCT of women with obesity and PCOS undergoing in vitro fertilisation compared liraglutide in addition to metformin with metformin alone [32]. When the pregnancy rate per embryo transfer cycles were compared, the combination was associated with a higher pregnancy rate compared to metformin alone (85.7% vs. 28.6%; *p* = 0.03). Of interest, weight loss between the groups was not statistically significant, 7.0 ± 6.0 kg and 7.5 ± 3.9 kg in the metformin groups and combination group, respectively, *p* = 0.246. This strengthens the hypothesis that GLP‐1 RAs may improve female fertility in pathways independent of weight loss. However, this was a pilot RCT with a sample size of 28 women and results need to be confirmed in larger studies. Interestingly, Zhao et al. recently conducted a study in women with PCOS and obesity to assess the effects of liraglutide on leptin promoter methylation in ovarian granulosa cells, where women underwent ovulation induction for cell collection. In an ad hoc analysis, the liraglutide group demonstrated significantly higher natural pregnancy rates during follow‐up compared to the metformin group (93.3% vs. 33.3%, *p* < 0.001), alongside improved menstrual cycle recovery (86.7% vs. 53.3%, *p* < 0.05). While pregnancy was not the primary outcome of this study, these findings further support the potential fertility benefits of GLP‐1 RAs [35].

Given the limitations of the currently available literature, future studies are needed to address these weaknesses by including larger samples, longer follow‐up and addressing bias in the design and analysis. The American Society for Reproductive Medicine report that the high dropout rate from RCTs assessing obesity and fertility in women presents a challenge for future prospective trials [49]. In this case, observational population‐based studies can help overcome these limitations by increasing the sample size and providing longer follow‐up. Additionally, the participants in most studies were relatively young, with an average age below 30 years. In a 2022 report by the Office for National Statistics, the majority of births in the UK were in women aged 30–34 years [50]. Therefore, the effect of weight‐lowering drugs on fertility in older age groups may differ.

This review comes with some strengths; one strength is that it adds to the limited evidence on the effect of weight‐lowering drugs on female fertility. Another strength is that it identifies the limitations of the current literature that need to be addressed in future studies. This review has several limitations. First, all identified clinical trials had small sample sizes. Second, some studies were assessed as having a high risk of bias, which limits the interpretation of our pre‐planned meta‐analyses. Third, all trials employed an open‐label design, potentially introducing detection bias. However, most studies utilised objective outcome measures, such as serum midluteal progesterone levels. Additionally, safety and cost outcomes were not assessed, as they were outside the predefined scope of this review; however, these represent important considerations for clinical decision‐making and should be addressed in future studies. Furthermore, four of the included studies were supported by academic or institutional funding, with no clear evidence of pharmaceutical industry sponsorship. However, three studies did not report funding sources, which limits the assessment of potential funding‐related bias. Finally, only one study evaluated a GLP‐1 RA, despite their increasing use for weight management.

## 5 Conclusion

This systematic review provides a summary of the literature on the impact of weight‐lowering drugs on natural fertility in women with overweight and obesity. Existing studies have primarily examined the effects of orlistat, while one recent study evaluated semaglutide combined with metformin. While in most studies orlistat was associated with improved ovulation rates when compared with lifestyle modifications, no significant difference was observed when compared with metformin. The study evaluating semaglutide showed higher natural pregnancy rates compared to metformin alone. However, these findings are limited by small sample sizes and methodological limitations. Data on conception, pregnancy and live birth rates are scarce. Future research should focus on conducting adequately powered, high‐quality studies in addition to investigating the effects of newer weight‐lowering drugs on clinically meaningful outcomes such as pregnancy and live birth rates.

## Author Contributions

S.J.A. was responsible for designing the review protocol, writing the protocol and manuscript, conducting the search, screening studies, assessing the quality of included studies, extracting and analysing data, interpreting results and updating reference lists. D.M.A. was responsible for screening studies, assessing the quality of included studies and reviewing and writing the manuscript. L.W. contributed to manuscript revision and writing. K.H. contributed to the design of the review protocol, arbitrating potentially eligible studies and revision and writing of the manuscript. R.B. was responsible for designing the review protocol, writing the protocol and manuscript, arbitrating potentially eligible studies, interpreting results and overseeing the review process. S.J.A. would like to acknowledge Hamad Medical Corporation, Qatar, which provided a scholarship for her studies.

## Funding

The authors have nothing to report.

## Ethics Statement

The authors have nothing to report.

## Conflicts of Interest

The authors declare no conflicts of interest.

## Supporting information


**Table S1:** Ovid MEDLINE (1946 to 16 October 2024).
**Table S2:** Embase Classic+ Embase (1947 to 2024 October 16).
**Table S3:** Cumulative Index to Nursing and Allied Health Literature.
**Table S4:** Cochrane Central Register of Controlled Trials.
**Table S5:** Clinicaltrials.gov.
**Figure S1:** Forest plot of the effect of orlistat versus lifestyle modifications on ovulation.

## Data Availability

All data generated or analysed during this study are included in this published article and its Supporting Information file.
