# Supplementary material for: Weight‐Lowering Drugs and Natural Female Fertility—A Systematic Review and Meta‐Analysis
Source: Clin Obes. 2026 Jun 17;16(4):e70092. doi: 10.1111/cob.70092 (PMC13274556; doi:10.1111/cob.70092)

Weight-Lowering Drugs and Natural Female Fertility– A Systematic Review and Meta-Analysis

Shaikha Jabor Alnaimi[1,2], Dana Muwafag Alsugeir[3], Li Wei[1], Kirsten Harvey[4], Ruth Brauer[1]

[1] Research Department of Practice and Policy, School of Pharmacy, University College London, London, United Kingdom

[2] Pharmacy Department, Hamad bin Khalifa Medical City, Hamad Medical Corporation, Doha, Qatar

[3] Department of Pharmacy Practice, College of Pharmacy, Imam Abdulrahman bin Faisal University, Dammam, Saudi Arabia

[4] Research Department of Pharmacology, School of Pharmacy, University College London, London, United Kingdom

Corresponding author: Ruth Brauer

Postal address: BMA House, Tavistock Square, London, WC1H 9JP, United Kingdom

Telephone: +442077535900

Email: r.brauer@ucl.ac.uk

Supplementary Material

Systematic Search

Table S1: Ovid MEDLINE (1946- 16 October 2024)

Date	Search	Search terms	Hits
1.10.2025	1	exp Obesity/ or exp Overweight/ or exp Adiposity/ or Body mass index/ or obes*.ti,ab. or overweight*.ti,ab. or body mass index.ti,ab. or BMI.ti,ab. or adiposity.ti,ab.	779697
1.10.2025	2	exp weight loss drugs/ or exp anti-obesity agents/ or exp Glucagon-like peptide-1 receptor agonists/ or weight loss drugs.ti,ab. or weight lowering drugs.ti,ab. or anti-obesity*.ti,ab. or antiobesity*.ti,ab. or Orlistat.ti,ab. or GLP-1 RA.ti,ab. or glucagon-like peptide-1 receptor agonists.ti,ab. or semaglutide.ti,ab. or Liraglutide.ti,ab. or Tirzepatide.ti,ab.	40814
1.10.2025	3	exp Fertility/ or exp pregnancy/ or exp infertility/ or exp preconception/ or exp conception/ or exp fecundity/ or exp live birth/ or exp reproduction/ or exp ovulation/ or exp in vitro fertilization/ or exp IVF/ or Fertility.ti,ab. or pregnan*.ti,ab. or infertility.ti,ab. or pregnancy rate.ti,ab. or preconception.ti,ab. or conception.ti,ab. or fecundity.ti,ab. or live birth.ti,ab. or reproduct*.ti,ab. or ovulation.ti,ab. or in vitro fertilization.ti,ab. or IVF.ti,ab.	1806834
1.10.2025	4	1 and 2 and 3	443
1.10.2025	5	Limit 4 to English	432
1.10.2025	6	(exp animal/ or exp invertebrate/ or animal experiment/ or animal model/ or exp plant/ or exp fungus/) not exp human/	5843238
1.10.2025	7	5 not 6	362

Table S2: Embase Classic+ Embase (1947 to 2024 October 16)

Date	Search	Search terms	Hits
1.10.2025	1	exp Obesity/ or exp Overweight/ or exp Adiposity/ or Body mass index/ or obes*.ti,ab. or overweight*.ti,ab. or body mass index.ti,ab. or BMI.ti,ab. or adiposity.ti,ab.	1587930
1.10.2025	2	exp weight loss drugs/ or exp anti-obesity agents/ or exp Glucagon-like peptide-1 receptor agonists/ or weight loss drugs.ti,ab. or weight lowering drugs.ti,ab. or anti-obesity*.ti,ab. or antiobesity*.ti,ab. or Orlistat.ti,ab. or GLP-1 RA.ti,ab. or glucagon-like peptide-1 receptor agonists.ti,ab. or semaglutide.ti,ab. or Liraglutide.ti,ab. or Tirzepatide.ti,ab.	91713
1.10.2025	3	exp Fertility/ or exp pregnancy/ or exp infertility/ or exp preconception/ or exp conception/ or exp fecundity/ or exp live birth/ or exp reproduction/ or exp ovulation/ or exp in vitro fertilization/ or exp IVF/ or Fertility.ti,ab. or pregnan*.ti,ab. or infertility.ti,ab. or pregnancy rate.ti,ab. or preconception.ti,ab. or conception.ti,ab. or fecundity.ti,ab. or live birth.ti,ab. or reproduct*.ti,ab. or ovulation.ti,ab. or in vitro fertilization.ti,ab. or IVF.ti,ab.	2471164
1.10.2025	4	1 and 2 and 3	1544
1.10.2025	5	limit 4 to English	1520
1.10.2025	6	(exp animal/ or exp invertebrate/ or nonhuman/ or animal experiment/ or animal tissue/ or animal model/ or exp plant/ or exp fungus/) not (exp human/ or human tissue/)	9401389
1.10.2025	7	5 not 6	1322

Table S3: Cumulative Index to Nursing and Allied Health Literature

Date	Search	Search terms	Hits
1.10.2025	1	TI ((MH "Body Mass Index") or (MH "Obesity+") or Body mass index or obes* or overweight or body mass index or BMI or adiposity) OR AB ((MH "Body Mass Index") or (MH "Obesity+") or Body mass index or obes* or overweight* or body mass index or BMI or adiposity)	285,468
1.10.2025	2	TI ((MH "Antiobesity Agents") or (MH "Glucagon-Like Peptide-1 Receptor agonists") or Weight lowering drugs OR weight loss drugs OR anti-obesity* OR antiobesity* OR Orlistat OR GLP-1 RA OR glucagon-like peptide-1 receptor agonist OR semaglutide OR Liraglutide OR Tirzepatide) OR AB ((MH "Antiobesity Agents") or (MH "Glucagon-Like Peptide-1 Receptor agonists") or Weight lowering drugs OR weight loss drugs OR anti-obesity* OR antiobesity* OR Orlistat OR GLP-1 RA OR glucagon-like peptide-1 receptor agonist OR semaglutide OR Liraglutide OR Tirzepatide)	8,575
1.10.2025	3	TI ((MH "Fertilization in Vitro") or (MH "Ovulation") or (MH "Reproduction") or (MH "Pregnancy") or (MH "Infertility") or (MH "Fertility") or fertility OR pregnan* OR infertility OR pregnancy rate OR preconception OR conception OR fecundity OR live birth OR reproduct* OR ovulation OR in vitro fertilization OR IVF) OR AB ((MH "Fertilization in Vitro") or (MH "Ovulation") or (MH "Reproduction") or (MH "Pregnancy") or (MH "Infertility") or (MH "Fertility") fertility OR pregnan* OR infertility OR pregnancy rate OR preconception OR conception OR fecundity OR live birth OR reproduct* OR ovulation OR in vitro fertilization OR IVF))	366,537
1.10.2025	4	1 and 2 and 3	101
1.10.2025	5	Limit to English	101

Table S4: Cochrane Central Register of Controlled Trials

Date	Search	Search terms	Hits
1.10.2025	1	MeSH descriptor: [Obesity] explode all trees	21795
1.10.2025	2	MeSH descriptor: [Overweight] explode all trees	25542
1.10.2025	3	MeSH descriptor: [Body Mass Index] explode all trees	14226
1.10.2025	4	(obes* or overweight or adiposity or body mass index or BMI):ti,ab,kw (Word variations have been searched)	135151
1.10.2025	5	1 or 2 or 3 or 4	135237
1.10.2025	6	MeSH descriptor: [Anti-Obesity Agents] explode all trees	1052
1.10.2025	7	MeSH descriptor: [Glucagon-Like Peptide-1 Receptor agonists] explode all trees	25
1.10.2025	8	MeSH descriptor: [Orlistat] explode all trees	349
1.10.2025	9	MeSH descriptor: [Liraglutide] explode all trees	1030
1.10.2025	10	(Weight lowering drugs OR weight loss drugs OR anti-obesity* OR antiobesity* OR Orlistat OR GLP-1 RA OR glucagon-like peptide-1 receptor agonist OR semaglutide OR Liraglutide OR Tirzepatide):ti,ab,kw (Word variations have been searched)	27776
1.10.2025	11	6 or 7 or 8 or 9 or 10	28017
1.10.2025	12	MeSH descriptor: [Pregnancy] explode all trees	34519
1.10.2025	13	MeSH descriptor: [Infertility] explode all trees	4594
1.10.2025	14	MeSH descriptor: [Pregnancy Rate] explode all trees	2469
1.10.2025	15	MeSH descriptor: [Fertilization] explode all trees	294
1.10.2025	16	MeSH descriptor: [Fertility] explode all trees	548
1.10.2025	17	MeSH descriptor: [Live Birth] explode all trees	512
1.10.2025	18	MeSH descriptor: [Reproduction] explode all trees	37092
1.10.2025	19	MeSH descriptor: [Fertilization in Vitro] explode all trees	2998
1.10.2025	20	(pregnan* OR fertility OR infertility OR pregnancy rate OR preconception OR conception OR fecundity OR live birth OR reproduct* OR ovulation OR in vitro fertilization OR IVF):ti,ab,kw (Word variations have been searched)	138513
1.10.2025	21	12 OR 13 OR 14 OR 15 OR 16 OR 17 OR 18 OR 19 OR 20	140309
1.10.2025	22	5 AND 11 AND 21	903

Table S5: Clinicaltrials.gov

Date	Search terms	Hits
1.10.2025	Fertility or pregnancy AND (Weight lowering drugs OR weight loss drugs OR anti-obesity OR antiobesity OR Orlistat OR GLP-1 RA OR glucagon-like peptide-1 receptor agonist OR semaglutide OR Liraglutide OR Tirzepatide)	43

Figure S1: Forest plot of the effect of orlistat versus lifestyle modifications on ovulation

Study or Subgroup	Orlistat Events	Orlistat Total	Control Events	Control Total	Weight	Risk Ratio IV, Random, 95% CI
Al-Qahwajy et al.	60	60	48	60	51.2%	1.25 [1.10, 1.42]
Kumar et al.	10	30	1	30	4.3%	10.00 [1.36, 73.33]
Munir et al.	18	30	1	15	4.6%	9.00 [1.32, 61.14]
Rahman et al.	37	60	27	60	39.9%	1.37 [0.97, 1.93]
**Total (95% CI)**		**180**		**165**	**100.0%**	**1.55 [1.01, 2.38]**
Total events	125		77			

Heterogeneity: Tau² = 0.09; Chi² = 8.37, df = 3 (P = 0.04); I² = 64%

Test for overall effect: Z = 2.00 (P = 0.05)

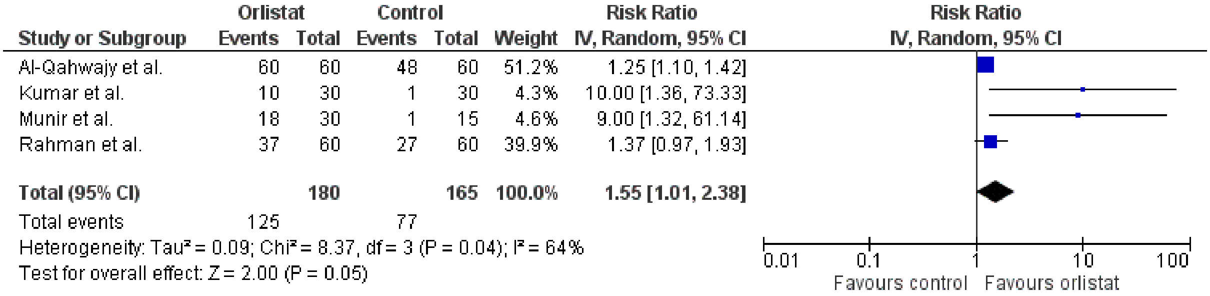

Supplement: Supplementary file 1 — Table S1: Ovid MEDLINE (1946 to 16 October 2024). Table S2: Embase Classic+ Embase (1947 to 2024 October 16). Table S3: Cumulative Index to Nursing and Allied Health Literature. Table S4: Cochrane Central Register of Controlled Trials. Table S5: Clinicaltrials.gov. Figure S1: Forest plot of the effect of orlistat versus lifestyle modifications on ovulation. [file COB-16-e70092-s001.pdf]
